# Swallowing Characteristics in Patients with Multiple System Atrophy Analyzed Using FEES Examination

**DOI:** 10.1007/s00455-023-10619-5

**Published:** 2023-09-21

**Authors:** Francesco Mozzanica, Nicole Pizzorni, Angelo Eplite, Daniela Ginocchio, Anna Colombo, Gabriele Mora, Federico Ambrogi, Tobias Warnecke, Antonio Schindler

**Affiliations:** 1 Department of Clinical Sciences and Community Health, University of Milan, Milan, Italy; 2 IRCCS Multimedica, Ospedale San Giuseppe, Milan, Italy; 3 Department of Biomedical and Clinical Sciences, University of Milan, Milan, Italy; 4 ALS Center, Istituti Clinici Scientifici Maugeri IRCCS, Milan, Italy; 5 Department of Neurology and Neurorehabilitation at the Klinikum Osnabrück, Academic Teaching Hospital of the Westfälische Wilhelms-University of Münster, Osnabrück, Germany

**Keywords:** Deglutition disorders, Multiple system atrophy, Deglutition, Endoscopy

## Abstract

Patients with multiple system atrophy (MSA) frequently experience dysphagia but only few studies analyzed its characteristics. The aim of this study was to describe the swallowing characteristics in these patients using fiberoptic endoscopic evaluation of swallowing (FEES). In addition, the swallowing abilities in patients with predominantly cerebellar MSA (MSA-C) and predominantly parkinsonian MSA (MSA-P) were compared. Twenty-five patients with MSA (16 MSA-P and 9 MSA-C) were enrolled. Clinical data including age, sex, functional oral intake scale (FOIS) score, body mass index (BMI) and the results of the global disability-unified MSA rating scale (GD-UMSARS) were collected. Three different textures of food (liquid, semisolid, solid) were provided during FEES examination. The characteristics of dysphagia (safety, efficiency, phenotype) and laryngeal movement alterations were analyzed. Delayed pharyngeal phase (92%) and posterior oral incontinence (52%) were the phenotypes more frequently seen. Penetration was more frequent with Liquid (68%), while aspiration occurred only with Liquid (20%). Residues of ingested food were demonstrated both in the pyriform sinus and in the vallecula with all the consistencies. Vocal fold motion impairment was the laryngeal movement alteration most frequently encountered (56%). No significant differences between patients with MSA-P and MSA-C in the dysphagia characteristics and laryngeal movement alterations were found. Patients with MSA frequently experience swallowing impairment and altered laryngeal mobility. Dysphagia characteristics and laryngeal movements alterations seems to be similar in MSA-C and MSA-P.

## Introduction

Multiple system atrophy (MSA) is an adult-onset, sporadic, progressive neurodegenerative disease caused by oligodendroglial aggregation of α-synuclein affecting predominantly the nigrostriatal, olivo-ponto-cerebellar, and autonomic systems [1–4]. MSA is characterized by varying severity of parkinsonian features, cerebellar ataxia, autonomic failure, urogenital dysfunction, and corticospinal disorders [1]. MSA progresses rapidly with a mean survival of 6 to 10 years after diagnosis [4]. The current criteria define four degrees of certainty for diagnosis, neuropathologically established MSA, clinically established MSA, clinically probable MSA, and possible prodromal MSA, and two phenotypes according to the clinical characteristics: predominantly cerebellar (MSA-C) or parkinsonian (MSA-P) [5].

In the recently developed criteria for MSA diagnosis [5], stridor, defined as respiratory sound caused by laryngeal dysfunction leading to glottal narrowing [6, 7], has been added as supportive non-motor feature. Laryngopharyngeal dysfunction is frequent and has been associated with decreased life expectancy and quality of life [1]. As far as the laryngeal dysfunction is concerned, Gandor et al. [1] analyzed laryngeal movement disorders using a specific task protocol and demonstrated their high prevalence in patients with MSA and suggested that irregular arytenoid cartilage movements could be used as a clinical marker to delineate MSA from PD with a high specificity and sensitivity. Dysphagia has been reported as a frequent and disabling symptom in MSA as well. However, despite its high prevalence (ranging from 31 to 78% [8]) and important clinical consequences such as aspiration pneumonia, sudden death due to aspiration, malnutrition, dehydration, and infectious complications [9–11], only few studies analyzed the swallowing characteristics in patients with MSA [12]. In particular, the dysphagia phenotype in this population has never been investigated. Scarce information regarding the differences between MSA-P and MSA-C in dysphagia features and severity are available; moreover, the majority of previous reports used videofluoroscopic swallowing study (VFSS) to assess dysphagia [13–19] rather than fiberoptic endoscopic evaluation of swallowing (FEES) [12], which allows a combined assessment of swallowing and laryngeal function. Consequently, few information regarding the swallowing characteristics analyzed through FEES are available so far [12, 20], even if severe dysphagia or the need for percutaneous endoscopic gastrostomy (PEG) for feeding are considered milestones of disease progression in MSA [8].

The aims of this study are: (1) to describe the dysphagia phenotype in MSA patients analyzed using FESS; (2) to compare the swallowing abilities in patients with MSA-C and MSA-P; (3) to evaluate laryngeal motility in patients with MSA; (4) to analyze the associations among the characteristics of dysphagia (safety and efficiency impairment, dysphagia phenotypes) and laryngeal motility alterations. The relevance of this study lies in the fact that a deeper knowledge of the characteristics of dysphagia in patients with MSA might be useful in the clinical practice for early diagnosis and proper dysphagia treatment.

## Material and Methods

The present cross-sectional study was conducted in accordance with the Declaration of Helsinki, and it was previously approved by the Institutional Review Board of our hospital. Data were collected prospectively.

### Participants

Participants underwent a thorough neurologic assessment and were diagnosed with either clinically established or probable MSA-P or MSA-C according to criteria proposed by the Movement Disorder Society [5]. Clinical data from a total of 25 patients (14 females and 11 males) affected by MSA (16 patients with MSA-P and 9 patients with MSA-C) were evaluated. All the enrolled subjects met the following inclusion criteria: age above 18 years; full oral diet with more than a single consistency; functional oral status (all natural teeth or partial tooth loss rehabilitated with an adjusted partial dental prosthesis [21]), no medical history of gastroenterological, respiratory, rheumatologic, metabolic, hematologic disorders. Exclusion criteria were intolerance to the components of the tested foods, additional neurologic diseases, history of head and neck cancer.

The functional oral intake scale (FOIS) [22–24], a seven-point ordinal scale indicating limitations in oral feeding which ranges from one (nothing by mouth) to seven (total oral diet with no restriction), was used to collect information regarding patient’s oral intake. The FOIS was administered immediately before the FEES examination. Information regarding the body mass index (BMI) were also collected as well as the results of the fourth part (IV, Global Disability Scale) of the unified MSA rating scale (GD-UMSARS) [25] as a marker of disease stage. Specifically, its scores range from 1 (completely independent. Able to do to all chores with minimal difficulties or impairment. Essentially normal) to 5 (totally dependent and helpless. Bedridden). The FOIS scale was selected because it has been validated in Italian [24] and it is widely used in dysphagic population. The GD-UMSARS Item 2 of Part 1 has not been used as it focuses on swallowing impairment, while the FOIS focuses on functional oral intake. In addition, according to the results of El Fassi et al. [26] the UMSARS-based assessment of dysphagia alone seems not to completely capture the key aspects of pharyngo-laryngeal dysfunction reflecting swallowing efficiency.

All the patients received exhaustive explanations regarding aims and objectives of the research, FEES evaluation and all the possible risks involved. All the enrolled patients gave their written informed consent. The characteristics of the enrolled patients are reported in Table 1.

**Table 1:** Characteristics of the enrolled population

Patient n°	MSA	GD-UMSARS	Sex	Age	FOIS	BMI
1	MSA-P	2	M	80	7	26.22
2	MSA-P	2	F	66	7	33.29
3	MSA-P	2	F	57	6	19.13
4	MSA-P	2	F	81	7	22.04
5	MSA-P	3	F	63	7	20.66
6	MSA-P	3	M	71	7	27.68
7	MSA-P	4	F	76	7	22.50
8	MSA-P	4	F	57	7	20,03
9	MSA-P	4	M	64	5	27.04
10	MSA-P	4	F	73	5	21.48
11	MSA-P	4	M	78	5	23.03
12	MSA-P	4	M	55	5	20.76
13	MSA-P	4	F	67	5	18.61
14	MSA-P	4	F	78	5	16.53
15	MSA-P	4	F	68	7	26.95
16	MSA-P	4	F	71	7	34.77
17	MSA-C	1	M	62	7	26.78
18	MSA-C	1	M	61	7	25.80
19	MSA-C	1	M	61	7	23.48
20	MSA-C	2	M	69	7	27.04
21	MSA-C	2	M	68	7	26.03
22	MSA-C	3	F	64	7	19.83
23	MSA-C	3	F	70	7	26.56
24	MSA-C	4	F	57	7	21.37
25	MSA-C	4	M	62	7	24.16

*MSA-P* predominantly parkinsonian multiple system atrophy, *MSA-C* predominantly cerebellar multiple system atrophy, *FOIS* functional oral intake scale, *GD-UMSARS* Global disability scale of the unified MSA rating scale

### FEES Examination

Each FEES was conducted by a senior Phoniatrician. A XION EF-N flexible endoscope with a diameter of 3.4 mm and a length of 320 mm (XION GmbH, Berlin, Germany) mounted on an EndoSTROB E camera (XION GmbH, Berlin, Germany) was used. All the videos were processed using the software Daisy Viewer 2.0 (INVENTIS srl, Padua, Italy) and stored in an anonymous form in.AVI format.

Each patient was seated on a comfortable chair, leaning back (between 75 and 90° approximately) with his arms on the armrests and keeping the head in neutral position to obtain the best posture for the examination. No local anesthetic drugs (e.g., lidocaine spray) were used in order not to alter pharyngo-laryngeal sensibility [27]. The endoscope was introduced into the widest nasal cavity and kept at a level just inferior to the uvula to maximize the field of view, including the larynx, the glossoepiglottic valleculae and the pyriform sinuses [28–30]. Three different textures of food were provided during FEES examination to evaluate swallowing:

- Liquid: room temperature skim milk (< 50 mPa·s at 50s^−1^ and 300s^−1^; International Dysphagia Diet Standardisation Iniatiative—IDDSI Level 0) [30] was used for thin Liquid trials.
- Semisolid: room temperature Crème Line vanilla pudding (Nutrisens Medical SAS, Francheville, France) (2583.3 ± 10.41 mPa·s at 50s^−1^ and 697.87 ± 7.84 mPa·s at 300s^−1^; IDDSI Level 4) was used for semisolid trials.
- Solid: a quarter and half of an 8-g dry biscuit (4 g per trial; IDDSI Level 7) were used for Solid trials.

FEES examinations were rated independently by three operators using the video files. All of them were speech and language therapists (SLTs) with at least 5 years of experience in FEES examinations. SLTs were blind to each other and to participants’ data, since videos were stored in an anonymous form. Two independent SLTs rated the videos using validated ordinal scales for swallowing safety and efficiency; inter-rater reliability between the 2 raters was analyzed. In case a difference > 1 level at each FEES rating scale occurred between the 2 raters, a 3rd SLT assessed the videos and decided on both ratings [27].

Different parameters were analyzed using the FEES examination:

- Dysphagia phenotypes defined according to the videoendoscopic scenarios proposed by Desuter [29, 31]. In particular, the presence of the following six phenotypes was assessed: protective deficit, posterior oral incontinence, delayed pharyngeal phase, oropharyngeal dyspraxia, propulsion deficit, resistive issue. Protective deficits include impairment of the following mechanisms: laryngeal elevation, glottis closure, tongue propulsion. Posterior oral incontinence is defined as inability of the patient to contain the bolus in the oral cavity when asked to. Delayed pharyngeal phase is defined as a delay of at least one on the following mechanisms when the patient is asked to swallow: arytenoid approximation and glottis closure, laryngeal elevation, tongue base propulsion, resulting in a progression of the bolus in the piriform sinuses beyond the glossopharyngeal ligaments before the swallowing reflex occurs. Oropharyngeal dyspraxia is the absence of pharyngeal swallowing and consequently retention of the bolus in the mouth or the appearance of cyclical movements of aborted movements of tongue base retraction. Propulsion deficit occurs when residue in the valleculae and/or the pirifom sinuses are found with weak tongue base retraction and/or, pharyngeal peristalsis and/or laryngeal elevation. Finally, resistive issue is found when residue occur in the retrocricoid region.
- Safety impairment (Penetration/aspiration): the severity of penetration/aspiration was rated using the Penetration Aspiration Scale (PAS) [32]. The PAS is an 8-points scale ranging from 1 (materials do not enter the airway) to 8 (materials enter the airway, passes below the vocal folds and no effort is made to eject). Penetration was defined as the bolus entering the laryngeal vestibule over the rim of the larynx (PAS score from 2 to 5). Aspiration was defined as the bolus passing below the true vocal folds (PAS score 6 or above). Safety of swallow was also evaluated similar to Tabor et al.’s study [33]. In particular, on the basis of the PAS score, each swallow was classified as unsafe if the material entered the laryngeal vestibule (PAS ≥ 3). In addition, in order to analyze the timing of unsafe swallows, each event was classified in “before”, “during” or “after” the swallow. The worst PAS score for each consistency and for each subject was considered for statistical analyses.
- Efficiency impairment (pharyngeal residue): the amount of pharyngeal residue after the swallow was rated using the yale pharyngeal residue severity rating scale (YPRSRS) vallecula and pyriform sinus [34]. Efficiency of swallow was also evaluated. In particular, a YPRSRS scores ≥ 3 (mild residue) was considered suggestive for an inefficient swallow. The worst YPRSRS score for each consistency and for each subject was considered for statistical analyses.
- Laryngeal movement analysis was performed using the MSA-FEES protocol used by Gandor et al. [1]. In particular, laryngeal assessment was performed at rest and during abductor and adductor tasks in order to evaluate the presence of 1 vocal fold (VF) motion impairment (VFMI); 2 VF fixation (VFF); 3 paradoxical VF motion (PVFM); 4 irregular arytenoid cartilages movements (iACM); 5 laryngeal stridor.

### Statistical Analysis

Statistical tests were performed using the SPSS 23.0 statistical software (SPSS Inc., Chicago, IL). The Kolmogorov–Smirnov test was used to test the normality of the distribution of FEES parameters among the patients. Since this test demonstrated that the distribution was not normal, non-parametric tests were used. Inter-rater reliability of FEES scoring between the two SLTs was analyzed. Weighted kappa with quadratic weighting was calculated [35]; k values were interpreted as follows: ≤ 0.20 poor agreement, 0.21–0.40 fair agreement, 0.41–0.60 moderate, 0.61–0.80 good, and 0.81–1.00 very good [36]. Mann–Whitney U test was used to compare the differences in age, BMI, FOIS scores between patients with MSA-P and MSA-C. Fisher’s exact test was used to compare the distribution of sex, GD-UMSARS score, dysphagia characteristics (safety, efficiency, phenotypes) and presence of laryngeal movement alterations between the two groups of patients because the variables were considered categorical [37, 38]. The Fisher’s exact test was also used to compare the distribution of patients according to the presence of unsafe swallow, inefficient swallow, and presence of laryngeal movement alterations on one side and the different dysphagia phenotypes leading to dysphagia on the other. Considering the number of tests performed in this last comparison, in order to avoid the risk of inflation of type 1 error a Bonferroni correction was applied, and a more stringent alpha level was used (p = 0.025).

## Results

Characteristics of the recruited population are reported in Table 1. There were 11 males and 5 females in the MSA-P group and 6 males and 3 females in the MSA-C group. This difference was found not significant at Fisher’s exact test (0.098). The median age was 68 and 63 years in the MSA-P and MSA-C group respectively. This difference was found not significant at Mann–Whitney test (p = 0.095). The median BMI was 23.1 and 25.9 in the MSA-P and MSA-C group respectively. Also, in this case the difference was found not significant at Mann–Whitney test (p = 0.522). No statistically significant differences in the distribution FOIS and GD-UMSARS scores were demonstrated at Fisher’s exact test between patients with MSA-P and MSA-C (p = 0.329 and p = 0.054 respectively). All the enrolled patients had a full oral diet and the median FOIS score was 7 (interquartile range 5–7). FEES protocol was performed using all the three consistencies in 19 patients, in the remaining 6 patients Solid was not tested, Liquid was not tested in 1 patient, and Semisolid was not tested in another 1 patient. One or more consistencies were not tested if there was a significant risk of choking. All the subjects included in the study completed the FEES protocol using at least one consistency. The time required to complete FEES never exceeded 15 min. FEES scoring inter-rater agreement ranged from good to very good. In particular, inter-rater agreement with each of the different consistencies for the PAS (k > 0.86) and for the YPRSRS in the vallecula and pyriform sinus (k > 0.81 and k > 0.85, respectively) was very good.

### Dysphagia Phenotypes

**Fig. 1:**
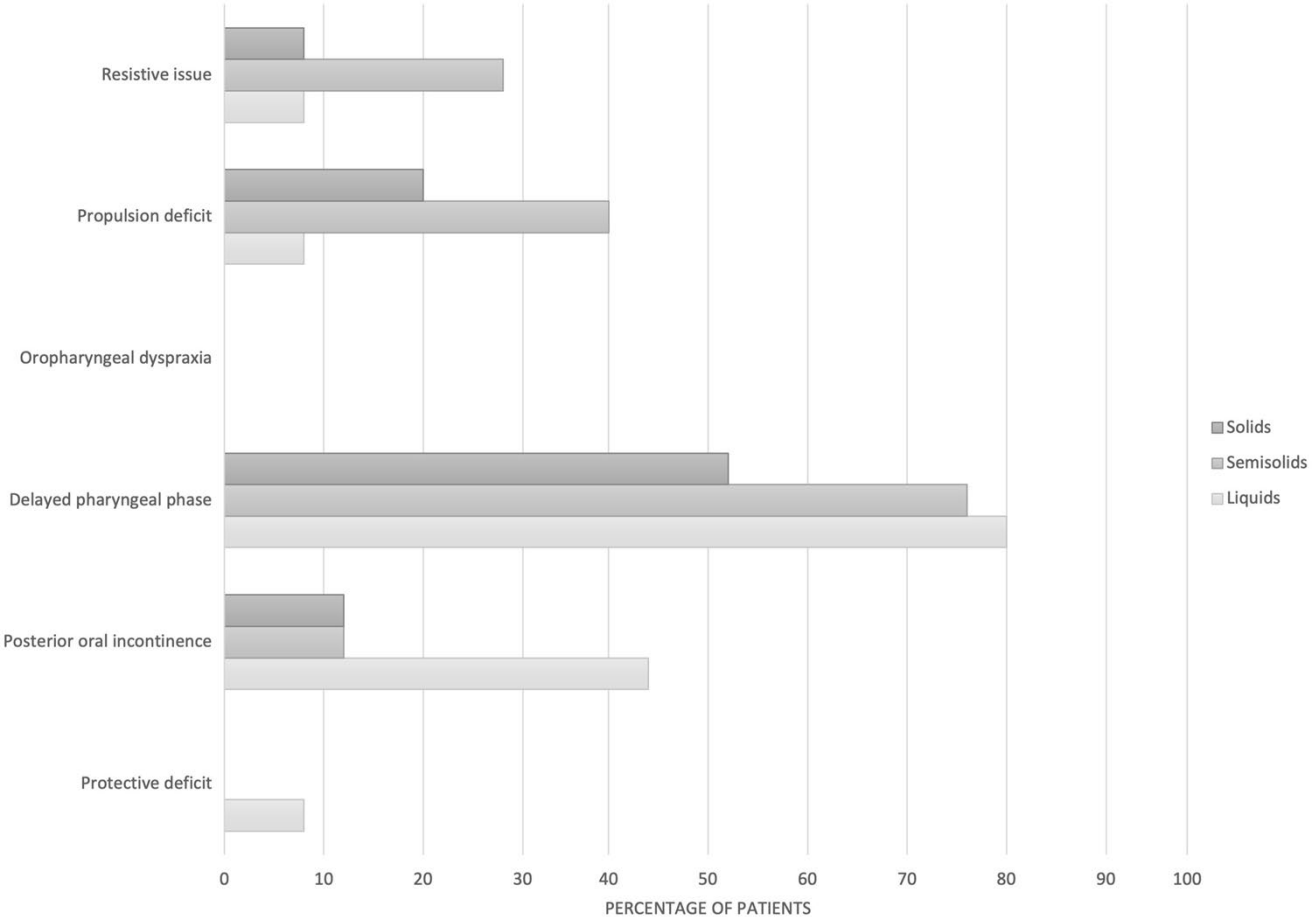
Pathophysiology of dysphagia for each consistency in the total cohort. The results are reported as percentages

**Fig. 2:**
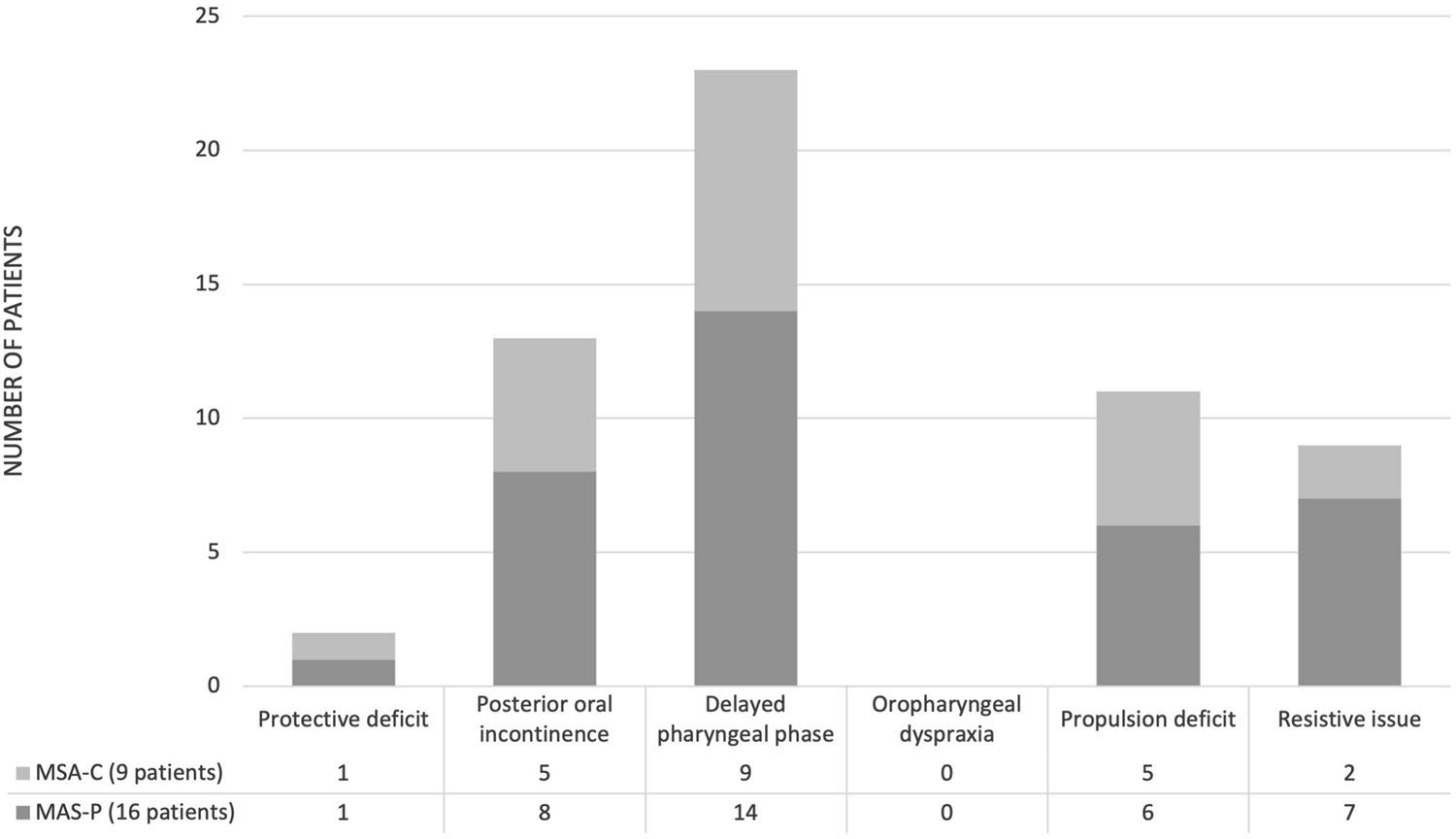
Pathophysiology of dysphagia within each diagnostic category. The results are reported as percentages. *MSA-P* predominantly parkinsonian multiple system atrophy, *MSA-C* predominantly cerebellar multiple system atrophy

The dysphagia phenotypes have been analyzed both in the overall sample for each consistency (Fig. 1) and in MSA-C and MSA-P subgroup of patients (Fig. 2). When considering the overall sample, a Delayed pharyngeal phase represented the most common phenotype (92%) with all consistencies, followed by Posterior oral incontinence mainly for Liquids (52%) and Propulsion deficit (44%) mainly with Semisolids and Solids. Five (20%) patients showed only one isolated phenotype, nine (36%) patients showed two combined phenotype, and eight (32%) patients showed three combined phenotypes. The differences between patients with MSA-P and MSA-C were not significant at Fisher test. In particular, for both groups Delayed pharyngeal phase and Posterior oral incontinence represented the most common phenotypes.

### Swallowing Safety

The differences in the distribution of the 3 PAS categories (normal, penetration, aspiration) for the 3 consistencies are reported in Table 2. Penetration was more frequent with Liquid (17 out of 24 patients, 68%), followed by Semisolid (13 out of 24 patients, 52%). Aspiration occurred only with Liquid (5 patients, 20%) and no aspiration was demonstrated for the Semisolid and Solid. The distribution of PAS categories between MSA-P and MSA-C patients was found different only for the Semisolid (p = 0.027) with penetration occurring significantly more frequently in patients with MSA-C. No statistically significant differences were found for the liquid and solid (p = 0.063 and p = 0.663 respectively).

**Table 2:** The results of the FEES examination obtained in the cohort of patients are reported

		Consistency								
		Liquid (24 patients)			Semisolid (24 patients)			Solid (19 patients)		
		Tot	MSA-P	MSA-C	Tot	MSA-P	MSA-C	Tot	MSA-P	MSA-C
PAS	Normal	2 (8%)	2 (12.5%)	0 (0%)	11 (44%)	10 (62.5%)	1 (6.25%)	11 (44%)	7 (43.8%)	4 (44.4%)
	Penetration	17 (68%)	12 (75%)	5 (55.6%)	13 (52%)	6 (27.5%)	7 (77.8%)	8 (32%)	5 (31.3%)	3 (33.3%)
	Aspiration	5 (20%)	1 (6.25%)	4 (44.4%)	–	–	–	–	–	–
YPRSRS vallecula		3 (1–3)	2 (2–3)	3 (2–3)	3 (1–4)	3 (2.5–5)	3 (2–4)	3 (1–4)	2.5 (2–4)	3 (1.5–3)
YPRSRS pyriform sinus		3 (1–4)	2 (2–3)	3 (3–4)	2 (1–5)	2 (1.5–4)	2 (1–4)	1 (1–4)	2 (1–4)	1 (1–3)

The results are reported as median and interquartile range (in brackets) for the ordinal data and as absolute (relative) frequencies for the categorical data. The PAS scores were categorized in Normal, Penetration (PAS score from 2 to 5) and Aspiration (PAS score 6 or above). Liquid consistency was tested in 24 patients, Semisolid consistency was tested in 24 patients, Solid consistency was tested in 19 patients.

*PAS* penetration aspiration scale, *YPRSRS* yale pharyngeal residue severity rating scale, *MSA-P* predominantly parkinsonian multiple system atrophy, *MSA-C* predominantly cerebellar multiple system atrophy

Regarding swallowing safety, with the Liquid consistency 16 out of 24 patients (66.7%) had unsafe swallows (5 patients had aspiration and 9 had penetration). Compromised airway protection occurred across all timing zones (before vs during vs after), however, unsafe swallow with Liquid consistency was more frequent “during” the swallow (10 out of 16 patients), followed by “before” the swallow (6 out of 16 patients). With the Semisolid consistency 5 patients (20.8%) had unsafe swallows but aspiration was never documented. Unsafe swallows with Semisolids occurred more frequent “during” swallow (3 out of 5 patients), followed by “before” swallow (2 out of 5 patients). Finally, with the Solid consistency unsafe swallows were demonstrated in 2 patients. Both patients had penetration “before” swallow. The distribution of unsafe swallows in the two groups of patients is reported in Table 3. No statistically significant differences between patients with MSA-P and MSA-C were demonstrated at Fisher test (p = 0.668, p = 0.555, and p = 0.386 for the Liquid, Semisolid, and Solid consistencies respectively).

**Table 3:** Airway safety profiles and efficiency of swallow expressed as absolute (relative) frequencies of patients with unsafe and inefficient swallows, stratified by consistency

	Consistency								
	Liquid			Semisolid			Solid		
	Total (%)	MSA-P (%)	MSA-C (%)	Total	MSA-P (%)	MSA-C (%)	Total (%)	MSA-P (%)	MSA-C (%)
Unsafe	16 (66.7)	10 (66.7)	6 (66.7)	5 (20.8)	3 (18.8)	2 (25.0)	2 (10.5)	2 (16.7)	0 (0.0)
Inefficient valleculae	13 (54.2)	7 (46.7)	6 (66.7)	16 (66.7)	11 (68.8)	5 (62.5)	10 (52.6)	6 (50)	4 (57.1)
Inefficient pyriform sinuses	16 (66.7)	7 (46.7)	9 (100)	11 (45.8)	8 (50)	3 (37.5)	4 (21.1)	2 (16.7)	2 (28.6)

Liquid consistency was tested in 24 patients, semisolid consistency was tested in 24 patients, solid consistency was tested in 19 patients.

*MSA-P* predominantly parkinsonian multiple system atrophy, *MSA-C* predominantly cerebellar multiple system atrophy

### Swallowing Efficiency

The YPRSRS scores obtained in patients with MSA-P, MSA-C are reported in Table 2. The median YPRSRS vallecula score was 3 for all the consistencies, while the YPRSRS pyriform sinus score was 3 for the Liquid, 2 for the Semisolid and 1 for the Solid. No statistically significant differences in the distribution of YPRSRS was demonstrated between the two groups with the only exception for the YPRSRS pyriform sinus score obtained during Liquid trials which appeared to be significantly lower in patients with MSA-P than in those with MSA-C (p = 0.451 at Fisher test).

Regarding swallowing efficiency, with the Liquid consistency 13 and 16 out of 24 patients (54.2% and 66.7%) had at least mild residue after swallow in the valleculae and pyriform sinuses respectively. With the Semisolid consistency 16 and 11 out of 24 patients (66.7% and 45.8%) had at least mild residue after swallow in the valleculae and pyriform sinuses respectively. Finally, with the Solid consistency 10 and 4 out of 19 patients (52.6% and 21.1%) had at least mild residue after swallow in the valleculae and pyriform sinuses respectively. The distribution of inefficient swallows in the two groups of patients is reported in Table 3. No statistically significant differences between patients with MSA-P and MSA-C were demonstrated at Fisher test for the valleculae region (p = 0.300, p = 0.553, and p = 0.570 for the liquid, semisolid, and solid consistencies respectively). Similarly, no statistically significant differences between the two groups were demonstrated at Fisher test for the region of pyriform sinuses for the Semisolid (p = 0.444) and Solid (p = 0.475) consistencies, while patients with MSA-C demonstrated a significant lower swallow efficiency with the Liquid consistency (p = 0.009).

### Laryngeal Movement Alterations

**Fig. 3:**
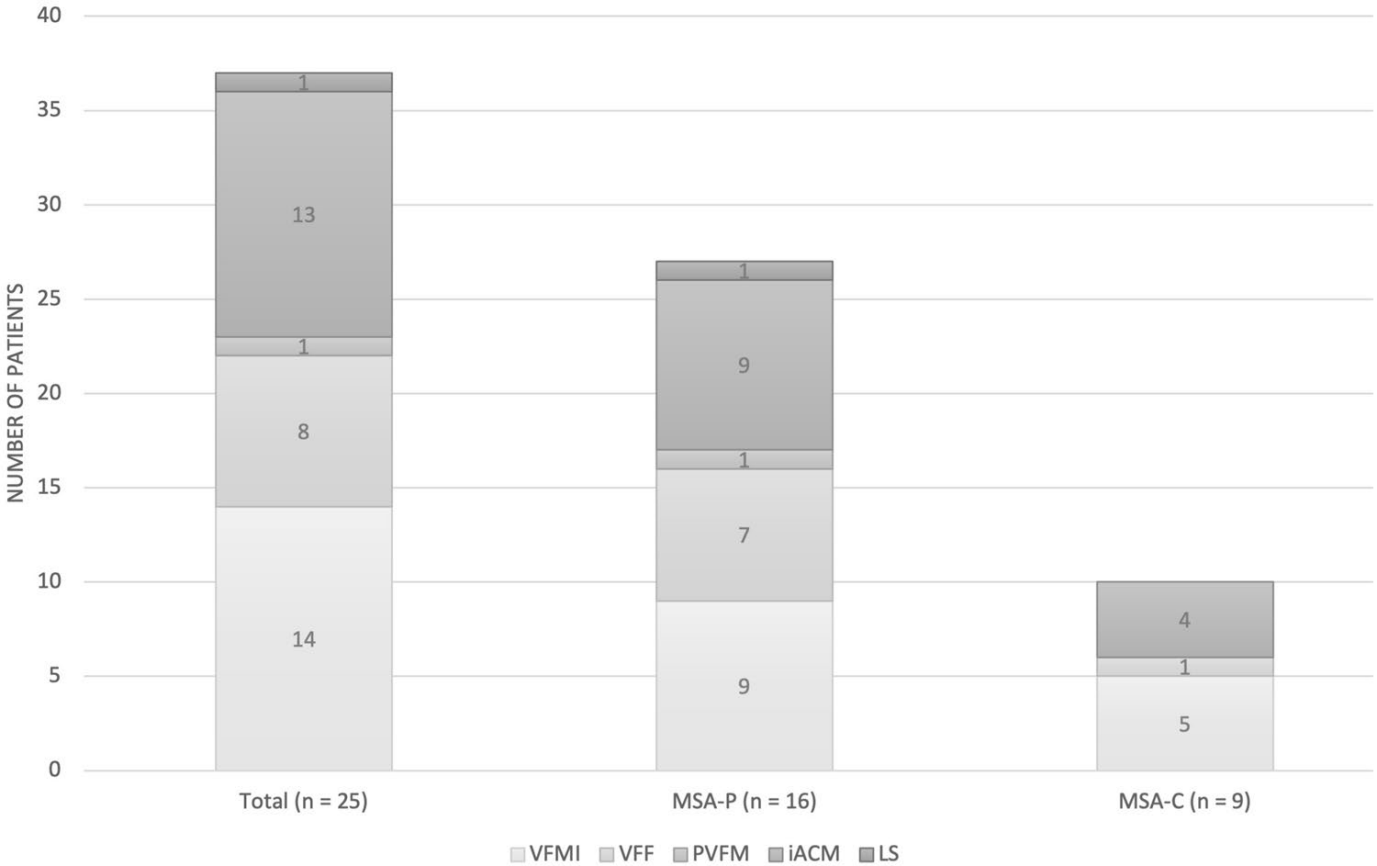
Laryngeal movement analysis in the cohort of patients. *VFMI* vocal fold motion impairment, *VFF* vocal fold fixation, *PVFM* paradoxical vocal fold motion, *iACM* irregular jitter and flutter of the arytenoid region, *LS* laryngeal stridor, *MSA-P* predominantly parkinsonian multiple system atrophy, *MSA-C* predominantly cerebellar multiple system atrophy. The number of patients affected by laryngeal movement disorders are reported

Laryngeal movement analysis was performed in all the enrolled subjects and the results are reported in Fig. 3. The majority of patients demonstrated at least 1 laryngeal movement alteration (19 out of 25 patients, 76%). VFMI and the iACM were the conditions most frequently encountered (14 patients, 56%, and 13 patients, 52% respectively). Six patients (24%) showed only one laryngeal movement alteration, 5 (20%) showed two combined conditions, 6 (24%) showed three combined conditions, and 2 (8%) showed four combined conditions. No statistically significant differences between patients with MSA-P and MSA-C at Fisher test were demonstrated.

### Association Analysis

The distribution of patients according to the presence of unsafe swallow, inefficient swallow and presence of laryngeal movement alterations on one side and the different dysphagia phenotypes on the other was studied using Fisher test. The results are reported in Table 4. The only significant difference were found for patients with Propulsion deficit who demonstrated a significant lower swallow efficiency (YPRSRS valleculae score) than patients without Propulsion deficit.

**Table 4:** The results of the Fisher test used to analyze the distribution of patients according to the safety and efficiency of swallow and the presence of laryngeal movement alterations on one side and the pathophysiological mechanisms leading to dysphagia on the other are reported

		Protective deficit	Posterior oral incontinence	Delayed pharyngeal phase	Propulsion deficit	Resistive issue
Unsafe	Liquid	0.435	0.235	0.667	0.156	0.553
	Semisolid	0.620	0.415	0.620	0.112	0.255
	Solid	0.895	0.386	0.895	0.263	0.456
Inefficient valleculae	Liquid	0.717	0.329	0.458	0.102	0.444
	Semisolid	0.435	0.536	0.565	**0.002**	0.332
	Solid	0.474	0.220	0.474	0.004	0.091
Inefficient pyriform sinuses	Liquid	0.435	0.556	0.667	0.156	0.553
	Semisolid	0.199	0.353	0.189	0.453	0.021
	Solid	0.789	0.525	0.788	0.033	0.373
Laryngeal movement alterations		0.260	0.582	0.260	0.570	0.560

In bold, statistically significant difference. According to the Bonferroni correction a p value of 0.025 was applied

## Discussion

In the present study the characteristics of dysphagia in patients with MSA were analyzed using FEES. The dysphagia phenotypes have been analyzed for the first time. In addition, the laryngeal motility, as well as the associations among unsafe and not-efficient swallow and laryngeal motility impairment were evaluated.

### Dysphagia Phenotypes

The large majority of patients with MSA demonstrated a swallowing impairment and in 80% of them 2 or more dysphagia phenotypes were detected. The more common were the Delayed pharyngeal phase with all the tested consistencies, followed by Posterior oral incontinence mainly visible with Liquids and Propulsion deficit with Semisolids and Solids. These data are in line with those of Warnecke et al. [20] who performed a systematic literature review in order to analyze the characteristics of neurogenic dysphagia using FEES. The authors reported that patients with atypical parkinsonian syndromes more commonly present pharyngolaryngeal movement disorders, premature bolus spillage and impaired swallowing reflex. Higo et al. [13] analyzed a total of 29 patients with MSA (22 MSA-C and 7 MSA-P) using VFS and found a delayed bolus transport in 73% of patients and impaired oral bolus control in 49%. The same author a couple of years later [14] analyzed the swallowing function in a group of 21 patients with MSA-C and found that swallowing function in the oral phase became gradually disturbed with the progression of the disease affecting both the bolus transport and bolus holding. Finally, Park et al. [15] who studied the swallowing outcomes following speech therapy in 7 patients with MSA-C using VFS found that patients suffered mainly from pharyngeal phase disturbances and premature bolus loss.

In our sample no differences between patients with MSA-C and MSA-P were demonstrated. This finding agrees with the results of Vogel et al. [12] who evaluated the endoscopic characteristics of dysphagia using FEES in 57 PD, 12 MSA-C and 45 MSA-P patients and found no differences in dysphagia pattern between MSA-C and MSA-P patients. In addition, Fernagut et al. [39] did not find any significant differences in the severity of dysphagia between patients with MSA-P and MSA-C. Lee et al. [18] analyzed the swallowing functions in a group of 31 patients with MSA-P and 21 with MSA-C using VFS. The authors did not find any significant differences in the oral and pharyngeal transit time, triggering of pharyngeal swallow, and premature bolus loss between the two groups. Moreover, Higo et al. [13, 14] demonstrated oral and pharyngeal dysfunction in both patients with MSA-P and MSA-C. The authors suggested that dysphagia in patients with MSA-P is a result of Parkinsonism which manifests with bradykinesia and rigidity of the tongue with consequent delayed bolus transport from the oral cavity to the pharynx and disturbance of bolus holding in the oral cavity, while in patients with MSA-C it is the disturbed coordination of tongue movement by cerebellar dysfunction, rather than Parkinsonism, which determines swallowing dysfunction in the oral phase at the early stage even if parkinsonism is also involved at the late stage [13, 14]. It is possible that in patients with MSA, both Parkinsonism and cerebellar dysfunction may distinctly contribute to dysphagia and consequently the absence of statistically significant differences between patients with MSA-P and MSA-C found in the present study might be related to the low number of enrolled patients. On the other hand, it is also possible that no difference exists in the dysphagia phenotypes in MSA-P and MSA-C. Further studies are needed to test these hypotheses.

### Swallowing Safety

Aspiration (bolus below the true vocal folds) occurred only with Liquid and was found in the 20% of the sample, while penetration (bolus enters the airway but not below the true vocal folds) was far more frequent (68%), in particular with Liquid, followed by Semisolid. Accordingly, safety of swallow was lower with Liquid with a compromised airway protection occurring both “during” and “before” the swallow in the majority of patients. These data suggest that the viscosity of the ingested food significantly affect the swallowing safety in patients with MSA. This finding agrees with Clavé et al. [40] who found that in patients with neurogenic dysphagia, increasing viscosity brought about a dramatic improvement on safety by minimizing penetration and aspiration during swallow.

As far as the high percentage of patients who demonstrated penetration and/or aspiration found in the present paper is concerned, the results here reported are in accordance with those of Lee et al. [18] who found that penetration or aspiration occurred in 67.8% of patients with MSA. Several previous studies evaluated the prevalence of dysphagia among patients with MSA but no information regarding the PAS score were provided. For example, Vogel et al. [12] by analyzing through FEES the swallowing abilities in a group of 57 patients with MSA found that penetration and aspiration occurred in 21% and 7% of the sample respectively.

The Fisher test did not demonstrate any significant differences in the distribution of PAS categories and safety of swallow between patients with MAS-P and MSA-C with the only exception for the Semisolid. In particular, penetration occurred significantly more frequently in patients with MSA-C. A possible explanation is related to a delayed pharyngeal phase which is particularly common even in early stages in patients with MSA-C as reported by Higo et al. [14]. However, no differences in the safety of swallow between the two groups was demonstrated thus suggesting that the severity of dysphagia is similar in both phenotypes. This finding agrees with those of Do et al. [17] who did not find any significant differences in the incidence of aspiration pneumonia between patients with MSA-P and MSA-C.

### Swallowing Efficiency

Residues of ingested food were demonstrated both in the pyriform sinus and in the vallecula with all the three consistencies. In addition, inefficient swallow was found in a high percentage of patients regardless of the consistency of the ingested food and of the phenotype of MSA, thus suggesting an impairment in the bolus propelling from the oropharynx to the esophagus. This finding is in accordance with the results of Higo et al. [13] who analyzed the manometric data of oropharyngeal and hypopharyngeal swallowing pressure in patients with MSA and found a decreased swallowing pressure compared to control subjects. In addition, the authors demonstrated bolus stasis at the pyriform sinuses in the 27.2% of patients with MSA. Similarly, Lee et al. [18] reported that the most common finding at VFS in patients with MSA was vallecular and pyriform sinus residues (89.8% and 63.2% respectively). Vogel et al. [12] found relevant pharyngeal residues in 50.9% of patients with MSA. Additionally, Ueha et al. [41] found abnormal hypertensive proximal esophageal contraction during swallowing, deficient upper esophageal sphincter (UES) relaxation and impaired UES relaxation in the 56%, 32% and 12% of patients with MSA.

### Laryngeal Movement Alterations

FEES examination revealed that most patients with MSA demonstrated at least one laryngeal movement alteration. IACM and VFMI were the conditions most frequently encountered; in addition, more than half of the enrolled patients demonstrated more than 2 laryngeal movement alterations. These results are in line to those previously reported even if the percentages of patients affected by laryngeal movement alterations appear lower than those reported by Gandor et al. [1] and by Warnecke et al. [20]. In the former study, the authors analyzed laryngeal movement in a group of 57 patients with MSA and found that iACM was the most prevalent laryngeal findings (91.2% of the sample) followed by VFMI (75.4%) [1]. In the latter, the authors found irregular arytenoid cartilages movements and vocal fold abduction restriction in all patients with MSA [20]. It is possible that the lower percentages found in our study might be related to differences in the severity of MSA among the patients enrolled in the different studies. In our sample 9 patients scored 1 or 2 at GD-UMSARS, in the study of Gandor et al. [1] the median Hoehn and Yahr stage was 4 while in the study of Warnecke et al. [20] the mean Hoehn and Yahr stage was 3.75. On the other hand, other authors reported a lower prevalence of laryngeal movement disorders in patients with MSA. Higo et al. [19] performed laryngoscopy on 38 MSA patients to assess laryngeal function and found VFMI in 17 of them. Simpson et al. [42] reported “flickering movements of the vocal folds” in 3 of 6 MSA patients during laryngoscopy. Irregular tremulous movement of the arytenoid cartilages was detected also by Shimohata et al. [43]. As suggested by Gandor et al. [1], the systematic assessment of laryngeal function using task provoking maximum VF movement (which allowed an easier identification of motion abnormalities) might explain the higher prevalence of laryngeal movement disorders found in this study.

The underlying pathology of laryngeal symptoms in MSA still remains under debate [1]. Nonetheless, iACM seems to predict the occurrence of glottic area reduction [44] and has been suggested as a valuable clinical marker for MSA allowing for delineation from Parkinson disease [1, 20]. Therefore, as proposed by Gandor et al. [1], an early evaluation of laryngeal function should be performed when MSA is suspected.

### Association Between Dysphagia Phenotypes and Laryngeal Functions

No significant differences in the safety of swallow and presence of laryngeal movement alterations on one side and the different dysphagia phenotypes on the other were demonstrated at Fisher’s exact test. This finding is difficult to compare since in none of the previous study the association among these elements was analyzed. On the other hand, patients with Propulsion deficit demonstrated a significantly lower swallow efficiency than patients without Propulsion deficit. This result agrees with those reported by Steele et al. [45]. In their systematic review the authors concluded that patients affected by poor pharyngeal contraction and tongue-base retraction experience more residues as the effort required for swallowing increases with thicker and harder foods.

### Study Limitations

There are several limitations in this study. First, the number of enrolled patients is quite small, even if in line with previous studies. Because of the rarity of the disease, it was difficult to collect a large number of patients. For this reason, the results here reported should be considered with caution. For example, even if there is no statistically significant difference between MSA-P and MSA-C for GD-UMSARS, it doesn't seem that the two groups are homogenous populations. In fact, according to a power calculation a difference of about 40% in the percentage of patients with GD-UMSARS less than 4, will have a power of 80% with alpha equal to 5% with 32 and 16 patients in MSA-P and MSA-C groups, respectively (hypothesizing a 2 to 1 ratio). This further stress the need to take with extreme caution the results of the comparison between patients with MSA-P and MSA-C. In addition, a larger number of enrolled patients would have allowed to perform subgroup analysis on the basis of the severity of the disease. Dysphagia phenotypes were judged as present or absent, according to the classification proposed by Desuter [31], whose psychometric properties still need to be analyzed.

In conclusion even in MSA patients under full oral nutrition with more than one consistency unsafe and inefficient swallow are quite frequent. The most common dysphagia phenotypes are delayed pharyngeal phase and posterior oral incontinence. Propulsion deficit is associated with lower swallow efficiency. Swallowing function and abnormal laryngeal movements seems to be similar in patients with MSA-C and patients with MSA-P.

## Declarations

### Research Involving Human and Animal Participants

The study was conducted in accordance with the Declaration of Helsinki, and it was previously approved by the Institutional Review Board of H. Sacco Hospital, Milan.
